# Ultrasound-based evaluation of plantar fascia and heel fat pad thickness in patients with type 2 diabetes: an observational cross-sectional study

**DOI:** 10.3389/fphys.2026.1835845

**Published:** 2026-07-09

**Authors:** Jorge Posada-Ordax, Marta Elena Losa-Iglesias, Eduardo Pérez-Boal, Roi Painceira-Villar, Bibiana Trevissón-Redondo, Josep Farradellas-Guitart, Antonio Javier Casanova-Malpica, Francisco Javier Ruiz-Sánchez, Daniel López-López, Klark Ricardo Becerro de Bengoa-Losa

**Affiliations:** 1 Department of Nursing and Physiotherapy, Universidad de León, Ponferrada, Spain; 2 Department of Nursing and Stomatology, Universidad Rey Juan Carlos, Alcorcón, Madrid, Spain; 3 FEBIO Research Group, Universidad Complutense de Madrid, Madrid, Spain; 4 HeQoL Research Group, Universidad de León, León, Spain; 5 Department of Podiatry, Faculty of Health Sciences, Universidad de Vitoria-Gasteiz, EUNEIZ, Vitoria-Gasteiz, Spain; 6 SALBIS Research Group, Universidad de León, León, Spain; 7 Department of Nursing, Faculty of Nursing, Physiotherapy and Podiatry, Universidad Complutense de Madrid, Madrid, Spain; 8 Research, Health and Podiatry Group, Department of Health Sciences, Faculty of Nursing and Podiatry, Industrial Campus of Ferrol, Universidade da Coruña, 15043 Ferrol, Spain; 9 Faculty of Economics and Business, Universidad Rey Juan Carlos, Madrid, Spain

**Keywords:** adipose tissue, aged, body composition, glycation end products advanced, risk factors

## Abstract

**Introduction:**

The plantar fat pad plays a key role in shock absorption and heel protection. In patients with controlled type 2 diabetes mellitus (cT2DM), advanced glycation end products (AGEs) may alter the structure of plantar tissue and affect its thickness and functionality. This study used ultrasound to compare the thickness of the plantar fat pad and plantar fascia between individuals with and without cT2DM and assessed the reliability of the ultrasound measurements.

**Methods:**

This prospective observational cross-sectional study examined 168 participants aged ≥60 years, including 84 with cT2DM and 84 without diabetes matched by sex. The plantar tissue thickness was measured in loaded and unloaded conditions using a portable ultrasound device. Intra-observer reliability was calculated using the intraclass correlation coefficient (ICC).

**Results:**

The ultrasound measurements showed excellent intra-observer reliability (ICC = 0.86–0.99). The groups showed no statistically significant differences in thickness of the heel plantar fat pad in loaded and unloaded conditions. However, the plantar fascia thickness showed significant differences between participants with diabetes and the control group in unloaded conditions (*p =* 0.040) and under load (*p =* 0.008). Differences between sexes were observed in height (*p <* 0.001) and weight (*p <* 0.001). Differences between the cT2DM group and the control group were also found in age (*p =* 0.001) and height (*p =* 0.010). After adjustment for age and height using linear regression analysis, the association between cT2DM and the plantar fascia thickness remained significant for only loaded conditions (*p =* 0.034).

**Discussion:**

Ultrasound demonstrated high intra-observer reliability, and the findings suggest a possible load-dependent association between cT2DM and plantar fascia thickness in the present study.

## 1 Introduction

The plantar fat pad is subcutaneous adipose tissue that occurs beneath the calcaneus and plays a fundamental role in dissipating impact forces and protecting heel structures (Jørgensen and Bojsen-Møller, 1989). This specialized adipose tissue consists of a more superficial layer of microchambers and a deeper layer of macrochambers (Taş, 2018). Thinning of these chambers is referred to as plantar fat pad atrophy (Latt et al., 2020; Balius et al., 2021), and multiple studies have demonstrated an association between this condition, heel pain, and poorer quality of life (Yi et al., 2011; Lopez-Lopez et al., 2019; Chang et al., 2022). The predisposing factors for this loss of adipose tissue include advanced age (Maemichi et al., 2024), variations in body weight (Ozdemir et al., 2004), metabolic diseases such as diabetes mellitus (Bus et al., 2004; Dalal et al., 2015; Yang et al., 2022), autoimmune diseases such as rheumatoid arthritis (Falsetti et al., 2004), and certain drugs such as corticosteroids (Vellingiri et al., 2022; Vali et al., 2024). In particular, in patients with diabetes mellitus, structural alterations in the plantar fat pad have been observed in relation to glycation processes (Monnier and Taniguchi, 2016).

Glycation is a spontaneous and non-enzymatic process in which reducing sugars such as glucose react with free amino groups in proteins, lipids, or nucleic acids. This process leads to structural modifications that can significantly compromise the function of the biomolecules involved. This phenomenon is also known as the Maillard reaction *in vivo* and is particularly relevant in situations of chronic hyperglycemia, which occurs in diabetes mellitus, or in physiological conditions associated with aging.

Over time, glycation gives rise to a heterogeneous group of compounds known as advanced glycation end products (AGEs), and their accumulation has been associated with a wide spectrum of chronic and degenerative pathologies, such as diabetic nephropathy, atherosclerosis, and the tissue stiffening that is characteristic of vascular and connective aging (Cho et al., 2007; Uceda et al., 2024). AGEs can induce protein cross-linking, loss of flexibility, aggregation, and functional impairment of the affected biomolecules. This process is not limited to the extracellular space, where it alters long-lived proteins such as collagen, but also occurs intracellularly, where reactive glycolytic by-products contribute significantly to the modification of cytosolic proteins, membrane components, and genetic material (Rowan et al., 2018).

In addition to their direct structural effects, AGEs are capable of activating intracellular signaling pathways by binding to specific receptors such as RAGE (receptor for advanced glycation end products). This interaction triggers a cascade of cellular responses that promote the production of proinflammatory cytokines. This positive feedback loop perpetuates glycation and amplifies tissue damage through chronic oxidative stress and persistent inflammation (Perrone et al., 2020). In individuals with diabetes, advanced glycation alters the collagen structure of the fibrous septa surrounding the fat lobules of the foot, which leads to a fragmented and disorganized arrangement of the fibers. This alteration caused by abnormal AGE-induced cross-linking reduces tissue flexibility and has been associated with thickening of the septal walls (approximately 10% to 25% greater thickness), an excess of structurally altered elastin, reduced compressive and cushioning capacity of the plantar fat pad, and a decrease in adipocyte size due to the thickening of these walls (Dalal et al., 2015). These AGE-induced collagen cross-linking processes may also contribute to structural remodeling and increased thickness of the plantar fascia.

Magnetic resonance imaging for the evaluation of plantar fat pad atrophy provides greater ability to detect soft tissue alterations in the plantar fascia and heel fat pad, but its clinical use is limited by high cost and the inability to perform dynamic real-time assessments (Draghi et al., 2017a). In this context, high-frequency musculoskeletal ultrasound has become an effective diagnostic tool for evaluating the plantar aspect of the foot as it combines a non-invasive approach with low cost, easy portability, real-time dynamic examination capability, and the possibility of bilateral comparison of both feet within the same session. These features make the technique particularly suitable for accurate and simultaneous measurements (Rogers and Cianca, 2010).

The primary objective of the present study was to use ultrasound imaging to compare the thickness of the plantar fat pad and plantar fascia in older patients with controlled type 2 diabetes mellitus (cT2DM) and in individuals without the disease. The reliability of the ultrasound measurements was also evaluated. The results could expand clinical knowledge regarding microstructural alterations of the plantar fat pad in patients with cT2DM, who are particularly susceptible to complications arising from mechanical overload and foot ulceration. Furthermore, by analyzing the reliability of ultrasound measurements in this context, this work supports the use of ultrasound as a non-invasive, accessible, and reproducible diagnostic tool for the preventive assessment and monitoring of plantar fat pad atrophy. This may facilitate the development of early detection protocols and personalized therapeutic strategies aimed at reducing the risk of complications associated with the diabetic foot.

The available evidence highlights the need for continued research on the biochemical mechanisms involved in AGE formation and their functional consequences in particularly susceptible tissues, such as those with high mechanical loading or low protein turnover, including the plantar fat pad. The study examines the research question of whether there are significant differences in the thickness of the heel plantar fat pad and plantar fascia between patients with and without cT2DM according to ultrasound, as well as whether this technique is sufficiently reliable for clinical use in detecting plantar fat pad atrophy. Based on the structural remodeling effects associated with AGEs, we hypothesized that patients with cT2DM would exhibit alterations in heel plantar fat pad thickness and increased plantar fascia thickness compared with non-diabetic individuals, and that ultrasound would be a reliable method for evaluating both structures.

## 2 Methods

This prospective, observational, descriptive, cross-sectional study was conducted at the University Podiatry Clinic of Ponferrada. A total of 168 participants participated, including 84 in the cT2DM group and 84 in the control group of individuals without diabetes. cT2DM was defined as a confirmed diagnosis of type 2 diabetes with HbA1c values below 7% (Bin Rakhis et al., 2022), treatment with oral antidiabetic medication and physical exercise. The sample was matched by sex to ensure homogeneity between groups. The inclusion criteria were age greater than 60 years, a lack of heel pain, a lack of current diabetes symptoms, and no diagnosis of plantar fasciitis within the previous year. The diabetes group had a confirmed diagnosis of type 2 diabetes mellitus made by an endocrinology specialist. This diagnosis was established according to internationally accepted criteria for diabetes mellitus (American Diabetes Association, 2010), which consider several clinical and biochemical criteria valid: glycated hemoglobin (HbA1c) above 6.5% (48 mmol/mol), fasting plasma glucose greater than or equal to 126 mg/dL (7.0 mmol/L) in at least two independent determinations, random plasma glucose of 200 mg/dL (11.1 mmol/L) or higher in the presence of characteristic symptoms of diabetes, or a value greater than 200 mg/dL (11.1 mmol/L) two hours after an oral glucose tolerance test. Additionally, patients were required to have a minimum disease duration of at least 10 years since diagnosis.

Participants were excluded if they had renal failure, musculoskeletal disorders, peripheral vascular disease, diabetic neuropathy, previous diabetic foot ulcers, or rheumatic diseases. Patients were also excluded if they had lower-limb amputations, Charcot foot, abnormal gait patterns, limb length discrepancies, deformities that could alter gait biomechanics, chronic corticosteroid use, a history of local corticosteroid injections in the heel, type 1 diabetes or any history of diabetes other than type 2, or long-term smoking habit. The study was conducted in accordance with the Declaration of Helsinki and the principles of human experimentation (World Medical Association, 2013). The design, development, and reporting of results followed the STROBE (Strengthening the Reporting of Observational Studies in Epidemiology) guidelines for observational studies (von Elm et al., 2007; Cuschieri, 2019). Ethical approval was granted by the Ethics Committee of the University of León (Reference: ÉTICA-ULE-142-2025).

### 2.1 Sample size

The required sample size was calculated using the difference between two independent groups and G*Power 3.1.9.2 software. The calculation was based on the Mann–Whitney *U* test with a normal distribution, two-tailed hypothesis, large effect size of 0.85, *α* error probability of 0.05, *β* level of 20%, desired power analysis of 80% (1−*β* error probability), and an allocation ratio (*N2*/*N1*) of 1. The minimum calculated sample size was 48 participants (24 per group), but a total of 168 participants were ultimately included to increase the statistical robustness. Participants were recruited from the University Podiatry Clinic (Ponferrada, Spain) using a consecutive non-randomized sampling method.

### 2.2 Equipment and materials

Ultrasound images were obtained using a high-frequency linear transducer (L14-6Ns) with an operating range of 6.0–14.0 MHz, which is suitable for the assessment of superficial structures. Examinations were performed with a portable Mindray^®^ M6 ultrasound system (Mindray Bio-Medical Electronics Co., Ltd., China) with a laptop-style configuration that was manufactured in 2012.

### 2.3 Study protocol

Ultrasound image acquisition and measurements were performed by a single podiatric clinician with more than 10 years of professional experience in musculoskeletal ultrasound and ongoing training. All measurements for each participant were carried out during a single session to minimize intra-observer variability. The same examiner conducted all ultrasound assessments to ensure standardization of the procedure and measurement reliability (Carr et al., 2021). Images were acquired using a standardized ultrasound configuration and a linear transducer with a working frequency of 10 MHz, gain adjusted between 70 and 85 dB, and a single focal zone positioned at depths between 0.5 and 2 cm.

An initial clinical interview was performed, the inclusion and exclusion criteria were verified, and written informed consent was obtained. Anthropometric variables were then recorded, including weight (kg), height (cm), and sex, and the body mass index (BMI) was calculated. Participants underwent ultrasound examination and assessment of the heel fat pad and plantar fascia after assuming a prone position on an examination table with the feet extending beyond the edge and maintained in a neutral position. No dorsiflexion of the tibiotalar joint was applied in order to avoid potential alterations in plantar fascia and fat pad measurements related to the calcaneal–Achilles–plantar system (Blackman et al., 2009; Ryskalin et al., 2024). Only the right foot was evaluated as bilateral comparison was not considered to provide clinically relevant information in the absence of clinical abnormalities, asymmetries, or morphological differences in the lower limbs after strict application of the exclusion criteria. In asymptomatic individuals without structural abnormalities, inter-limb differences in plantar tissues are generally minimal and not considered clinically relevant, which supports the use of unilateral assessment in this study (Karabay et al., 2007; Chen et al., 2021).

**Figure 1:**
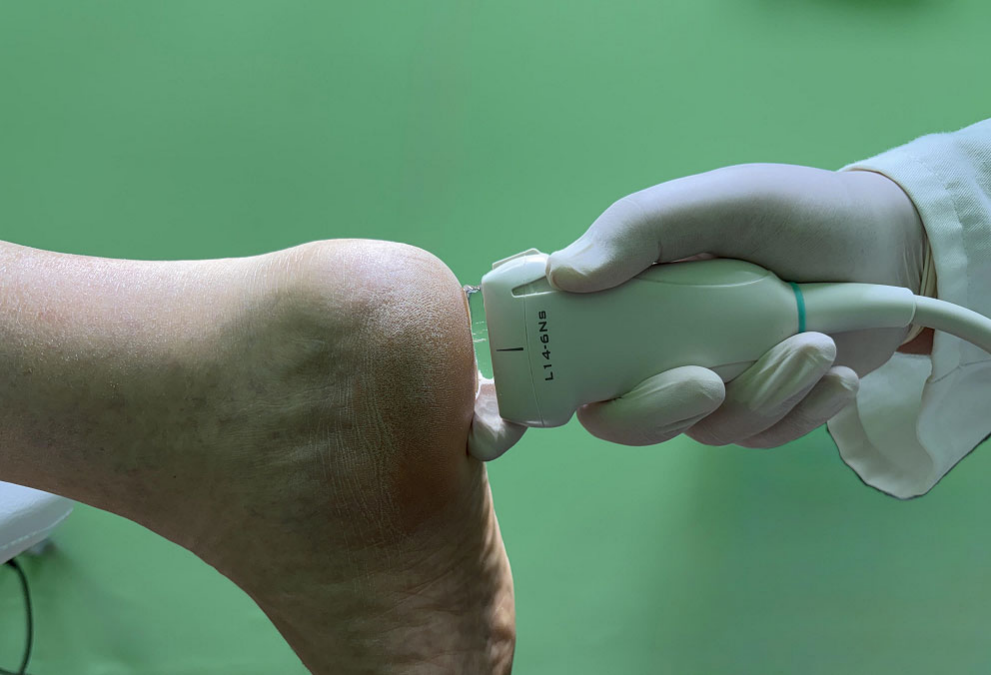
Ultrasound image acquisition using the “gel stand-off” technique, in which a generous amount of ultrasound gel is applied between the transducer and the skin to enable measurements without applying compression to the underlying tissues.

A previously described protocol was followed for ultrasound imaging of the heel (Morrison et al., 2021). The transducer was positioned over the medial calcaneal tubercle (**Figure 1**), oriented so that its longitudinal axis was aligned with the second ray of the foot and kept perpendicular to the skin surface. Fat pad measurements were performed at the point of maximum calcaneal prominence located centrally and equidistant from the lateral margins of the image. Thickness was assessed independently while excluding the plantar fascia to avoid overestimation of the soft tissue thickness.

Previous studies have highlighted the risk of combined measurements between the plantar fascia and the heel fat pad, so it was considered methodologically more appropriate to analyze both structures independently. For the measurement of the plantar fascia, the maximum thickness observed at its proximal insertion on the calcaneus was recorded using a longitudinal plane. This measurement site was selected in accordance with previously described ultrasound protocols as it is considered the most reproducible location for assessing fascial thickness (Analay et al., 2025).

**Figure 2:**
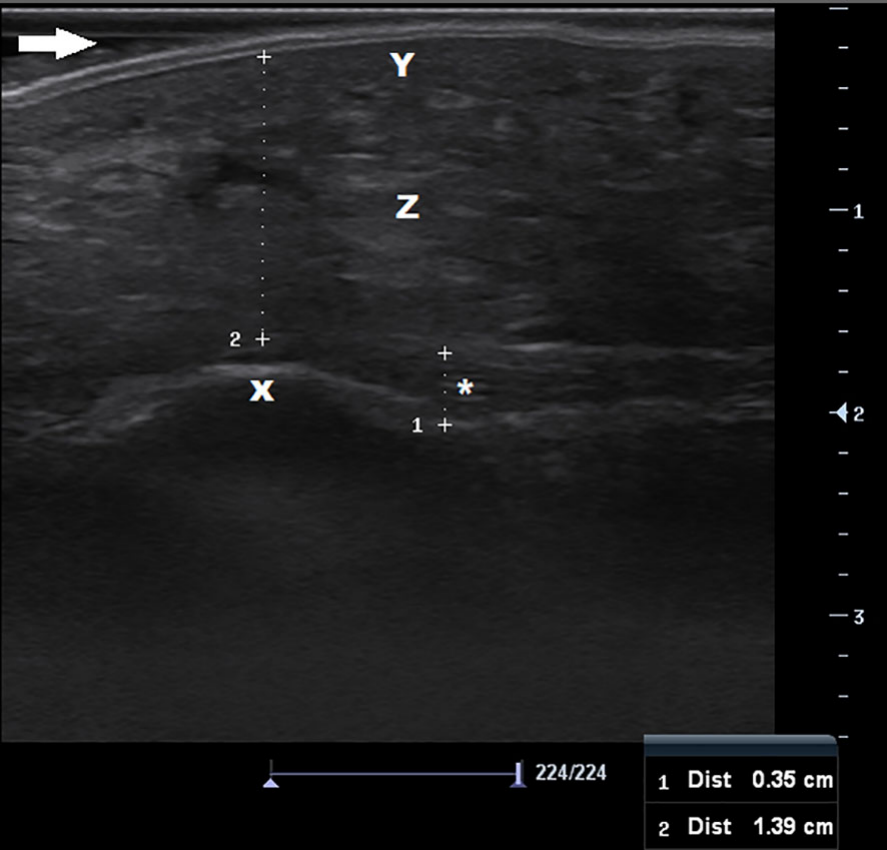
Ultrasound image acquisition under unloaded conditions. “X” indicates the point of maximum calcaneal convexity used as a reference for measuring the plantar fat pad (measurement 1), excluding the plantar fascia. Measurement 2 corresponds to the thickness of the plantar fascia. The asterisk (*) marks the measurement site. “Y” corresponds to the microchambers of the plantar fat pad, while “Z” corresponds to the macrochambers. The white arrow indicates the layer of ultrasound gel for the “gel stand-off” technique.

**Figure 3:**
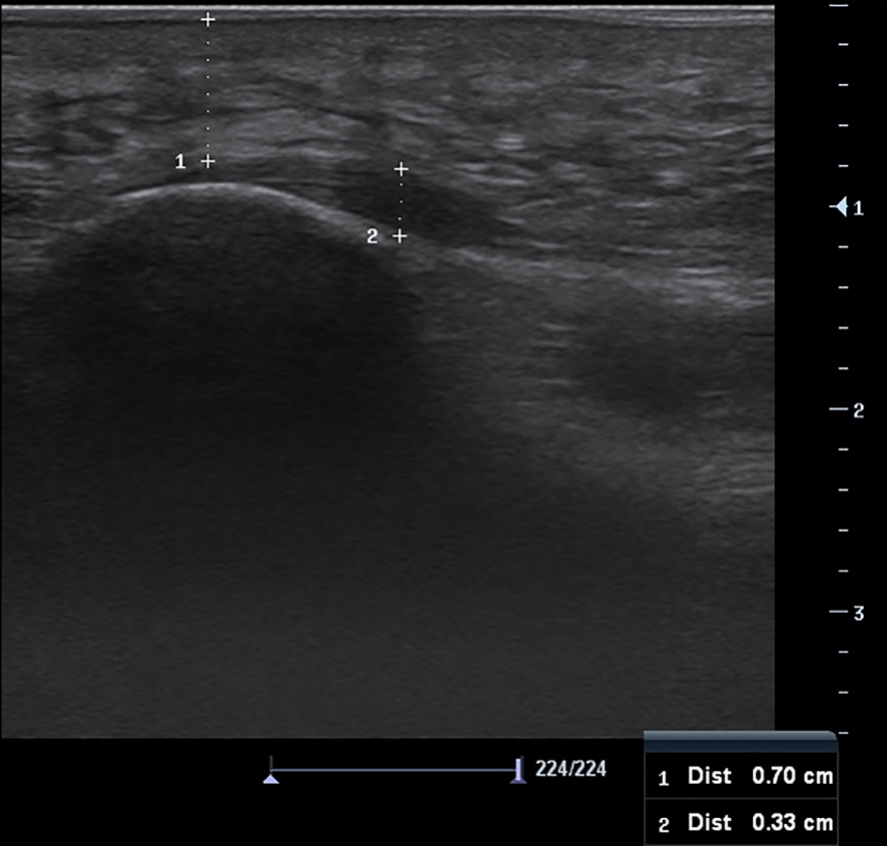
Ultrasound image under loaded conditions. Measurement 1 corresponds to plantar fat pad thickness, while measurement 2 corresponds to plantar fascia thickness at its proximal insertion on the calcaneus.

Unloaded measurements were performed using the “gel stand-off” technique, which consists of placing a thick layer of ultrasound gel between the skin and the transducer to avoid direct pressure on the evaluated structure (**Figure 2**) (Diamantopoulos et al., 2011; Corvino et al., 2020). For loaded measurements, progressive pressure was applied with the transducer until the observed structures no longer showed morphological changes, indicating full adaptation to the applied compression (**Figure 3**). All measurements were performed with three consecutive repetitions to standardize the pressure applied to the assessed region, increase measurement reliability, and reduce recording variability. Finally, the mean thickness values of the plantar fascia and plantar fat pad were calculated for statistical analysis.

### 2.4 Statistical analysis

The intra-observer reliability of the measurements was assessed by calculating the intraclass correlation coefficient (ICC) from three consecutive measurements performed under each condition (loaded and unloaded) as a statistical indicator of reliability (Crofts et al., 2014; Ribeiro et al., 2022). To interpret the ICC values, the categories established by Landis and Koch (Landis and Koch, 1977) were used as a reference: ≤0.20: poor; 0.21–0.40: fair; 0.41–0.60: moderate; 0.61–0.80: substantial; >0.81: almost perfect. According to the recommendations of Portney and Watkins (Portney and Watkins, 2017), clinical measurements with an ICC greater than 0.90 were considered reliable to increase the likelihood that results are consistent.

Normality of the data distributions was assessed using the Kolmogorov–Smirnov test. Quantitative variables were described as the mean and standard deviation (SD) with the 95% confidence interval. Student’s *t*-test for independent samples was applied to compare demographic characteristics and measurements between groups. The comparisons were performed with stratification by sex (female and male). Statistical significance was set at *p <* 0.05, data analysis was conducted using SPSS for Windows (version 22.0; SPSS Inc., Chicago, IL, USA).

## 3 Results

The intra-session intra-observer reliability results showed very high ICC values for all ultrasound measurements, including both plantar fat tissue and plantar fascia thickness under loaded and unloaded conditions. In particular, the ICCs were close to unity (ICC = 0.86–0.99), indicating excellent measurement consistency. In addition, the standard error of measurement (SEM) and minimal detectable change (MDC) were very low, indicating minimal measurement error and high precision in the repeatability of intra-observer evaluations (**Table 1**).

**Table 1:** Intra-observer reliability and measurement of ultrasound-based plantar fat pad and plantar fascia thickness measurements at the heel.

Variable	Mean (SD)	CI 95%	CV (%)	ICC (2,1) (CI 95%)	SEM	MDC	95% Normality values
Fat unloaded	1.68 (0.29)	(1.64 – 1.73)	17.26	0.99 (0.98 – 0.99)	0.02	0.08	(1.11 – 2.24)
Fascia unloaded	0.19 (0.07)	(0.18 – 0.20)	36.84	0.86 (0.82 – 0.89)	0.02	0.07	(0.05 – 0.32)
Fat loaded	1.05 (0.27)	(1.01 – 1.09)	25.71	0.99 (0.99 – 0.99)	0.02	0.07	(0.52 – 1.57)
Fascia loaded	0.18 (0.06)	(0.17 – 0.19)	33.33	0.99 (0.99 – 0.99)	0.00	0.01	(0.06 – 0.29)

ICC, intraclass correlation coefficient; CV, coefficient of variation; SEM, standard error of measurement; MDC, minimal detectable change; SD, standard deviation. CI, confidence interval. All thickness values are expressed in centimeters (cm).

The study included a total of 168 participants with equal sex distribution (84 women and 84 men). The mean age was 69.42 ± 11.89 years for women and 70.69 ± 11.38 years for men, while their mean heights were 157.23 ± 7.95 cm and 168.54 ± 9.04 cm, their mean body weights were 69.42 ± 15.49 kg and 81.40 ± 18.83 kg, and their mean body mass indexes were 28.30 ± 6.59 kg/m² and 28.72 ± 6.20 kg/m², respectively. Women and men showed statistically significant differences in height (*p <* 0.001) and body weight (*p <* 0.001) (**Table 2**).

**Table 2:** Demographic and anthropometric characteristics of the total sample according to sex.

Variable	Total mean ± SD (CI 95%)	Female mean ± SD (CI 95%)	Male mean ± SD (CI 95%)	P value (student’s t-test)
Age (years)	70.05 ± 11.62 (68.30 – 71.81)	69.42 ± 11.89 (66.88 – 71.97)	70.69 ± 11.38 (68.25 – 73.12)	P = 0.483
Height (cm)	162.89 ± 10.21 (161.34 – 164.43)	157.23 ± 7.95 (155.53 – 158.93)	168.54 ± 9.04 (166.61 – 170.48)	P < 0.001*
Body Mass (kg)	75.41 ± 18.21 (72.66 – 78.17)	69.42 ± 15.49 (66.11 – 72.74)	81.40 ± 18.83 (77.37 – 85.43)	P < 0.001*
BMI (kg/m^2^)	28.51 ± 6.38 (27.54 – 29.48)	28.30 ± 6.59 (26.89 – 29.72)	28.72 ± 6.2 (27.39 – 30.04)	P = 0.679

Values are shown as mean ± standard deviation (SD), with 95% confidence intervals shown in parentheses (CI). The Student’s t-test was used for statistical analysis, with the significance level set at P < 0.05. BMI, body mass index. * Statistically significant at p < 0.05.

Participants with and without cT2DM showed statistically significant differences in age (*p =* 0.001) and height (*p =* 0.010) (**Table 3**), as well as plantar fascia thickness under both loaded (*p =* 0.008) and unloaded (*p =* 0.040) conditions. No significant differences were found in plantar fat tissue thickness in any of the evaluated conditions (**Table 4**). When comparing control women versus women with cT2DM or control men versus men with cT2DM, no statistically significant differences were found in plantar fat tissue or plantar fascia measurements in either loaded or unloaded conditions (**Table 5**). Likewise, no statistically significant differences were observed in plantar fat tissue or plantar fascia thickness under either loaded or unloaded conditions when comparing men and women within the same group (**Table 6**).

**Table 3:** Comparison of demographic and anthropometric characteristics between participants with and without diabetes.

Variable	Control group Mean ± SD (CI 95%)	Diabetic group mean ± SD (CI 95%)	P value (student’s t-test)
Age (years)	67.19 ± 12.62 (64.49 – 69.89)	72.92 ± 9.78 (70.83 – 75.02)	P < 0.001*
Height (cm)	164.90 ± 9.37 (162.89 – 166.91)	160.88 ± 10.66 (158.60 – 163.16)	P = 0.010*
Body Mass (kg)	74.5 ± 17.60 (70.73 – 78.26)	76.33 ± 18.86 (72.29 – 80.36)	P = 0.515
BMI (kg/m^2^)	27.60 ± 6.44 (26.23 – 28.98)	29.42 ± 6.23 (28.08 – 30.75)	P = 0.066

Values are shown as the mean and standard deviation, with the 95% confidence interval in parentheses (CI). The Student’s t-test was used for statistical analysis, with the significance level set at P < 0.05. Participants were compared both between groups (diabetic vs. control). BMI, body mass index. * Statistically significant at p < 0.05.

**Table 4:** Comparison of plantar fat and fascia load/unload measurements between diabetic and non-diabetic subjects.

Variable	Control group mean ± SD (CI 95%)	Diabetic group mean ± SD (CI 95%)	P value (student’s t-test)
Fat unloaded	1.70 ± 0.28 (1.64 – 1.76)	1.66 ± 0.30 (1.60 – 1.73)	P = 0.407
Fascia unloaded	0.17 ± 0.06 (0.16 – 0.19)	0.20 ± 0.07 (0.18 – 0.21)	P = 0.040*
Fat loaded	1.07 ± 0.26 (1.01 – 1.13)	1.04 ± 0.27 (0.98 – 1.10)	P = 0.478
Fascia loaded	0.17 ± 0.06 (0.16 – 0.18)	0.20 ± 0.07 (0.18 – 0.22)	P = 0.008*

Values are expressed as mean ± standard deviation (SD), 95% confidence Interval (CI). The Student’s t-test was used for statistical analysis, with the significance level set at P < 0.05. All thickness values are expressed in centimeters (cm). * Statistically significant at p < 0.05.

**Table 5:** Comparison of plantar fat and fascia load/unload measurements between same sex in control and diabetes groups.

Variable	Female		P value (student’s t-test)	Male		P value (student’s t-test)
	Control group mean ± SD (CI 95%)	Diabetic group mean ± SD (CI 95%)		Control group mean ± SD (CI 95%)	Diabetic group mean ± SD (CI 95%)	
Fat unloaded	1.75 ± 0.30 (1.66 – 1.84)	1.72 ± 0.33 (1.62 – 1.82)	P = 0.709	1.65 ± 0.26 (1.57 – 1.74)	1.60 ± 0.26 (1.53 – 1.68)	P = 0.391
Fascia unloaded	0.18 ± 0.07 (0.16 – 0.20)	0.19 ± 0.05 (0.18 – 0.21)	P = 0.346	0.17 ± 0.05 (0.15 – 0.19)	0.20 ± 0.08 (0.18 – 0.23)	P = 0.058
Fat loaded	1.09 ± 0.28 (1 – 1.17)	1.07 ± 0.28 (0.99 – 1.16)	P = 0.817	1.05 ± 0.25 (0.97 – 1.13)	1 ± 0.25 (0.93 – 1.08)	P = 0.424
Fascia loaded	0.17 ± 0.06 (0.15 – 0.19)	0.19 ± 0.05 (0.17 – 0.21)	P = 0.053	0.17 ± 0.05 (0.15 – 0.19)	0.20 ± 0.08 (0.17 – 0.23)	P = 0.071

The values are shown as the mean and standard deviation (SD), with the 95% confidence interval in parentheses (CI). The Student’s t-test was used for statistical analysis, with the significance level set at P < 0.05. Participants were compared both between groups (diabetic vs. control) and between sexes. All thickness values are expressed in centimeters (cm).

**Table 6:** Comparison of plantar fat and fascia load/unload measurements between sex.

Variable	Female	Male	P value (student’s t-test)	Female	Male	P value (student’s t-test)
	Control group mean ± SD (CI 95%)	Control group mean ± SD (CI 95%)		Diabetic group mean ± SD (CI 95%)	Diabetic group mean ± SD (CI 95%)	
Fat unloaded	1.75 ± 0.30 (1.66 – 1.84)	1.65 ± 0.26 (1.57 – 1.74)	P = 0.392	1.72 ± 0.33 (1.62 – 1.82)	1.60 ± 0.26 (1.53 – 1.68)	P = 0.314
Fascia unloaded	0.18 ± 0.07 (0.16 – 0.20)	0.17 ± 0.05 (0.15 – 0.19)	P = 0.058	0.19 ± 0.05 (0.18 – 0.21)	0.20 ± 0.08 (0.18 – 0.23)	P = 0.061
Fat loaded	1.09 ± 0.28 (1 – 1.17)	1.05 ± 0.25 (0.97 – 1.13)	P = 0.424	1.07 ± 0.28 (0.99 – 1.16)	1 ± 0.25 (0.93 – 1.08)	P = 0.694
Fascia loaded	0.17 ± 0.06 (0.15 – 0.19)	0.17 ± 0.05 (0.15 – 0.19)	P = 0.072	0.19 ± 0.05 (0.17 – 0.21)	0.20 ± 0.08 (0.17 – 0.23)	P = 0.097

The values are shown as the mean and standard deviation (SD), with the 95% confidence interval in parentheses (CI). The Student’s t-test was used for statistical analysis, with the significance level set at P < 0.05. Participants were compared both between sexes. All thickness values are expressed in centimeters (cm).

**Figure 4:**
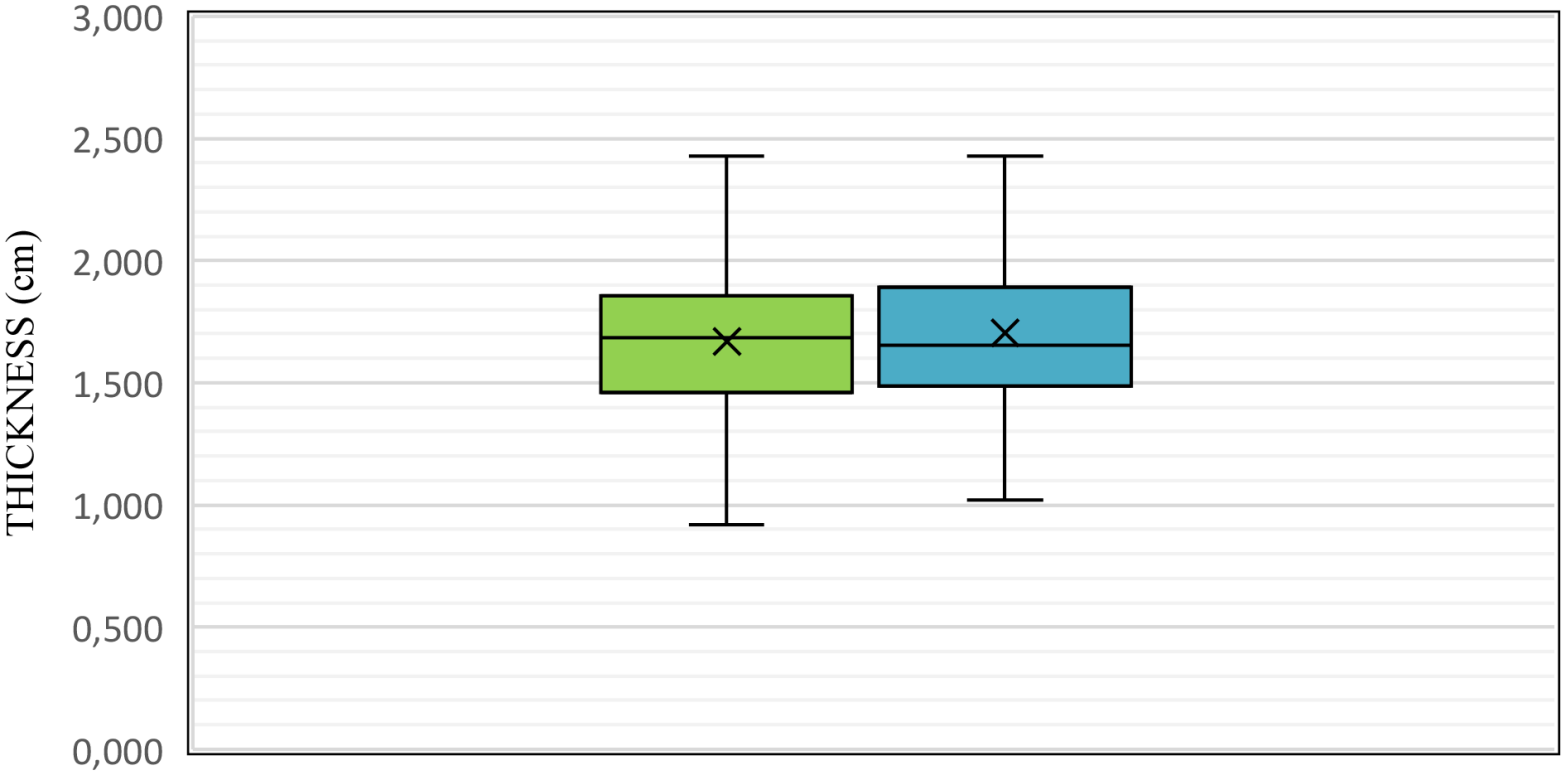
Boxplot of plantar fat pad thickness (unloaded condition) in diabetic and control groups. Green boxes indicate diabetic participants, and blue boxes indicate controls. The median, interquartile range, and mean (X) are shown.

**Figure 5:**
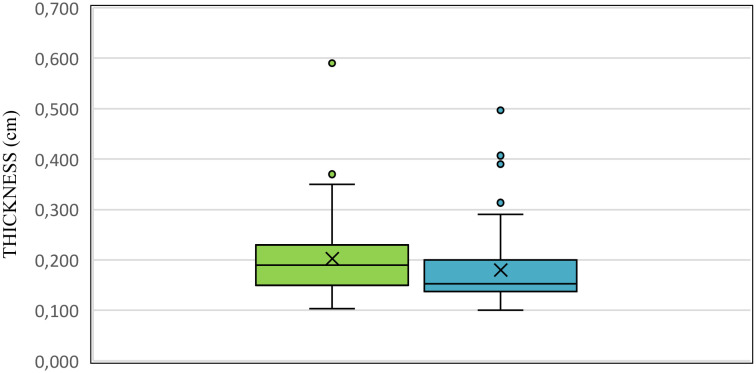
Boxplot of plantar fascia thickness (unloaded condition) in diabetic and control groups. Green boxes indicate diabetic participants, and blue boxes indicate controls. The median, interquartile range, mean (×), and outliers are shown.

**Figure 6:**
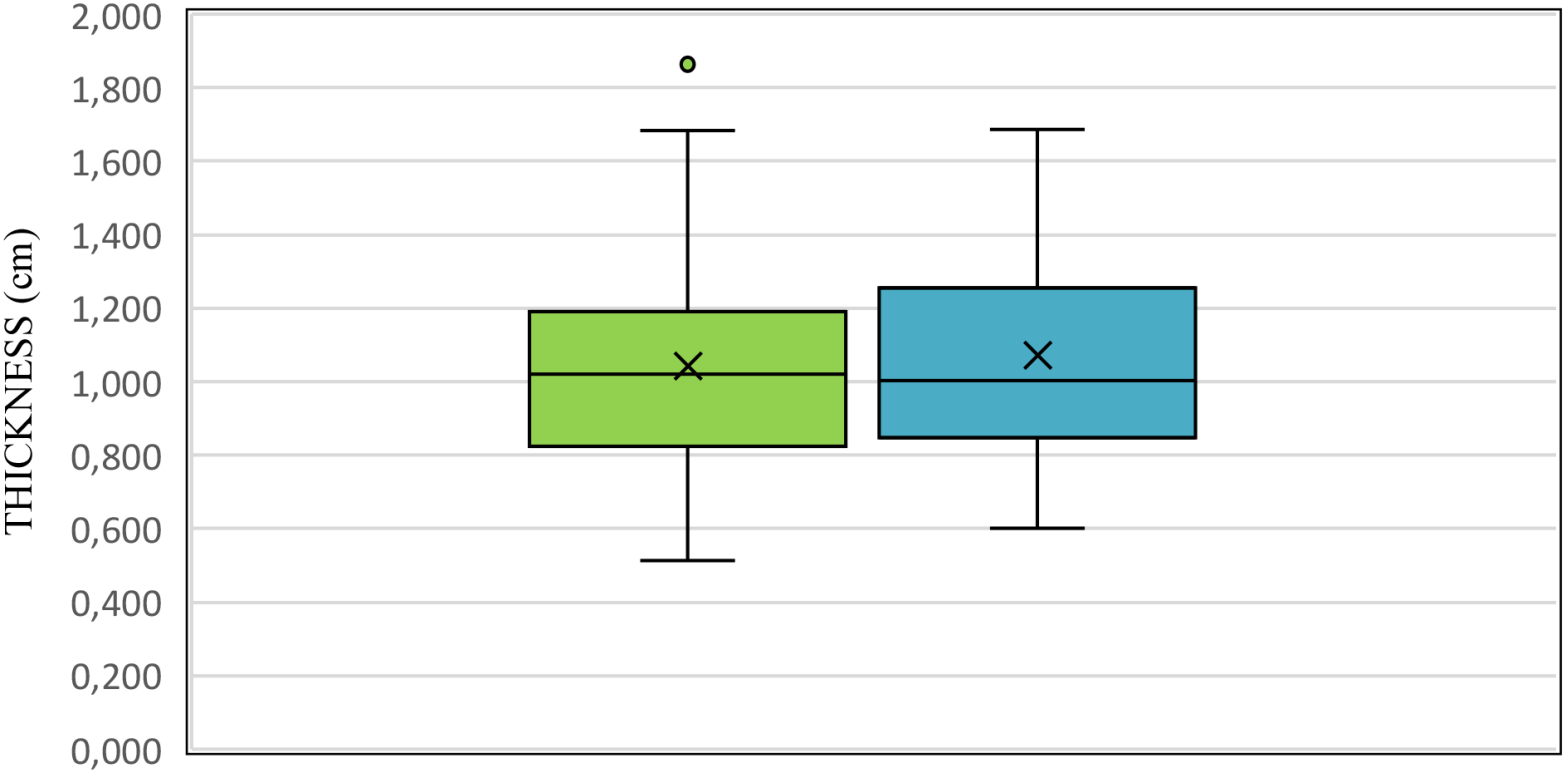
Boxplot of plantar fat pad thickness (loaded condition) in diabetic and control groups. Green boxes indicate diabetic participants, and blue boxes indicate controls. The median, interquartile range, mean (×), and outliers are shown.

**Figure 7:**
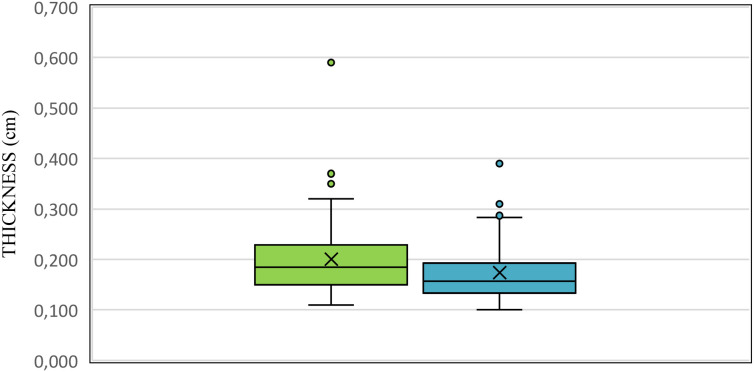
Boxplot of plantar fascia thickness (loaded condition) in diabetic and control groups. Green boxes indicate diabetic participants, and blue boxes indicate controls. The median, interquartile range, mean (×), and outliers are shown.

Additional linear regression analyses were performed to evaluate the potential influence of age and height on plantar fascia thickness (**Tables 7**, **8**). After adjustment, a statistically significant association between cT2DM and plantar fascia thickness was observed only under loaded conditions (*p =* 0.034), whereas no significant association was found under unloaded conditions. Neither age nor height were significant predictors in the adjusted models. **Figures 4**–**7** illustrate the distribution of plantar fat pad and plantar fascia thickness under loaded and unloaded conditions in both the diabetic and control groups. No relevant differences were observed in plantar fat pad thickness between groups in either condition (**Figures 4**, **6**). In contrast, the plantar fascia thickness was greater in the diabetic group than the controls in both loaded and unloaded conditions (**Figures 5**, **7**), and the difference was more pronounced under load.

**Table 7:** Linear regression analysis of plantar fascia thickness under unloaded conditions.

Variable	β coefficient	P value
Diabetes	0.0188	P = 0.102
Age (years)	0.0004	P = 0.412
Height (cm)	-0.0004	P = 0.531

Linear regression model evaluating the association between diabetes, age, and height with plantar fascia thickness under unloaded conditions. β: regression coefficient. Statistically significant at P < 0.05.

**Table 8:** Linear regression analysis of plantar fascia thickness under loaded conditions.

Variable	β coefficient	P value
Diabetes	0.0229	P = 0.034*
Age (years)	0.0005	P = 0.268
Height (cm)	-0.0004	P = 0.496

Linear regression model evaluating the association between diabetes, age, and height with plantar fascia thickness under loaded conditions. β: regression coefficient. * Statistically significant at p < 0.05.

## 4 Discussion

Ultrasound has been used to evaluate the thickness of plantar fat tissue and the plantar fascia in both unloaded and compressed conditions. The results demonstrated high intra-observer reliability of the ultrasound measurements in assessing plantar fat tissue thickness with high ICCs, which support the consistency of the protocol. These findings are consistent with previous studies that have also reported excellent reliability for this type of measurement. In a large sample of healthy participants, ICC values of 0.98 for the deep layer (macrochamber) and 0.92 for the superficial layer (microchamber) were obtained using a standardized ultrasound protocol under unloaded conditions and by averaging three consecutive measurements per region (Maemichi et al., 2020). Similarly, an ICC of 0.82 was reported for high-frequency ultrasound measurements in a study involving a large sample of patients with chronic plantar heel pain (Rogers et al., 2023). A recent study analyzed the functional characteristics of the heel fat pad in young, middle-aged, and older individuals and reported ICC values greater than 0.90 for both tissue layers under unloaded conditions and partial loading, which is consistent with the high reliability values observed in our study (Maemichi et al., 2024).

Another investigation assessed the reliability of ultrasound measurements of the Achilles tendon, plantar fascia, and heel fat pad in patients with hindfoot pain and analyzed measurements obtained with and without pressure. Two independent observers performed measurements according to a standardized protocol. That study reported intra-observer ICC values ranging of 0.89 to 0.96 without pressure and 0.88 to 0.97 with pressure for the fat pad, as well as high inter-observer reliability (ICC between 0.81 and 0.91 for a single measurement and up to 0.95 when averaging two measurements per examiner) (Johannsen et al., 2016). In the present study, it was only possible to assess intra-observer reliability, but the findings further support the robustness of ultrasound as a reliable tool for evaluating plantar tissue. Overall, the consistency of the results and their agreement with previous research suggest that ultrasound is a reliable method for assessing the morphology of both plantar fat tissue and the plantar fascia when applied using standardized and reproducible protocols.

The available evidence suggests that type 2 diabetes is not consistently associated with a significant reduction in heel fat pad thickness, particularly in patients with well-controlled disease. Several studies have reported an absence of morphological differences between individuals with type 2 diabetes and healthy controls, indicating that diabetes-related alterations may more consistently affect the mechanical properties of the tissue rather than the thickness measured using ultrasound (Hsu et al., 2000; Chatzistergos et al., 2014). However, studies have observed a significant decrease in fat pad thickness in patients with more advanced or poorly controlled diabetes, particularly in the presence of neuropathy and ulceration. This observation supports the hypothesis that subcutaneous tissue atrophy may contribute to plantar tissue deterioration and the development of complications (Gooding et al., 1985).

It has been shown that even in the absence of changes in total thickness, relevant internal mechanical alterations may occur, including modified deformation of the superficial microchambers and increased stiffness of the deeper macrochambers. These alterations are likely related to collagen glycation processes and fibrotic remodeling of the septa (Hsu et al., 2009). Similarly, it has been suggested that plantar tissue thickness may remain stable or even decrease in the early stages of diabetic neuropathy, whereas in more advanced stages, the thickness may increase, which reinforces that morphological alterations are more evident in contexts of long disease duration or poor metabolic control (Thomas et al., 2003). In this regard, the absence of differences in fat pad thickness may coexist with internal structural changes characterized by smaller fat globules and thickened fibrous septa, as well as a trend of progressively increasing stiffness.

Groups with diabetes have been reported to exhibit the greatest overall tissue stiffness, which supports the idea that diabetes primarily modifies the biomechanical behavior of the tissue rather than its absolute thickness (Tong et al., 2003). Consistent with this evidence, the results of the present study did not show statistically significant differences in heel plantar fat pad thickness between participants with controlled type 2 diabetes and controls in either loaded or unloaded conditions. This finding supports the hypothesis that early alterations may manifest more in mechanical terms or internal structural remodeling than as quantifiable morphological changes in tissue thickness. The findings suggest that reductions in plantar fat pad thickness may represent a later-stage complication of diabetes rather than an early alteration in well-controlled patients (Dalal et al., 2015; Bus et al., 2021; Bellomo et al., 2022).

Several studies have described a significant thickening of the plantar fascia in individuals with cT2DM than healthy controls, particularly in those who are overweight or obese. This finding has been attributed to both collagen glycation processes and increased mechanical stress associated with a higher BMI. Adiposity can increase loading on the soft tissues of the foot and joints and promote tissue remodeling even in the early stages of the disease. Furthermore, these changes may be accompanied by a reduction in stiffness according to elastography, suggesting that hyperglycaemia-induced effects may involve both structural alterations and modifications in tissue elasticity (Thomas et al., 2003; Saroha et al., 2023; Abate et al., 2012).

However, a significant reduction in plantar fascia thickness has also been reported in individuals with type 2 diabetes compared with controls, regardless of the presence of clinical neuropathy. This highlights the heterogeneity of findings and suggests that plantar structural changes may develop early in the course of the disease (Kumar et al., 2015). In this context, plantar fascia thickness has been proposed as a potentially useful measure of tissue glycation that could provide a non-invasive estimate of accumulated glycemic burden (Harish C. et al., 2022). The fascial thickening observed in type 2 diabetes may be explained by metabolic mechanisms, including non-enzymatic collagen glycation, extracellular matrix expansion secondary to fluid retention, and chronic low-grade inflammation. This would support connective tissue remodeling rather than a localized inflammatory process (Chincholikar et al., 2025). On the other hand, plantar fascia thickness has been shown to correlate positively with diabetes duration and HbA1c levels, suggesting that chronic hyperglycemia and the accumulation of AGEs, oxidative stress, low-grade inflammation, and microvascular alterations play a central role in structural tissue remodeling. These changes appear to be more pronounced in advanced stages or when complications are present (Bozkuş et al., 2025).

Consistent with this evidence, the present results initially showed statistically significant differences in plantar fascia thickness between participants with cT2DM and controls in both loaded and unloaded conditions. However, after adjusting for age and height through linear regression analysis, this association remained statistically significant only under loaded conditions. This suggests that the differences observed at rest may be influenced by other factors, while the association with diabetes remains significant under mechanical load.

Nevertheless, plantar fascia thickness should be interpreted as a non-specific ultrasound finding. Although the present study focused on patients with controlled type 2 diabetes mellitus and attempted to control for potential confounding factors through the inclusion and exclusion criteria, fascial thickening cannot be considered a diabetes-specific marker. Plantar fascia thickness may be influenced by local factors, such as plantar fasciitis (Mohseni-Bandpei et al., 2014a), local heel pain (Drake et al., 2022), or previous microtrauma (Radwan et al., 2016); systemic factors, such as obesity (Pappalardo et al., 2023), increased BMI (van Leeuwen et al., 2016), or aging (Abul et al., 2015); inflammatory conditions (Slouma et al., 2023); metabolic alterations, including diabetes-related changes or other disorders affecting connective tissue metabolism (Snedeker and Gautieri, 2014); and biomechanical factors, such as mechanical overload (Wearing et al., 2006), altered foot structure (Wearing et al., 2007), or changes in gait biomechanics (Phillips and McClinton, 2017).

From a clinical perspective, structural changes in the plantar fat pad, including fibrosis or disruption of its adipose architecture, may increase mechanical stress on the plantar fascia at its calcaneal insertion. In this context, a heel fat pad syndrome may coexist or even be misdiagnosed as plantar fasciopathy, potentially leading to additional complications such as microfissures within the fat pad. Therefore, both tissues should be considered when interpreting ultrasound findings and clinical symptoms (Ricci et al., 2024). The absence of differences in plantar fat pad thickness and the persistence of differences in plantar fascia thickness in only loaded conditions after adjustment suggest that structural alterations associated with type 2 diabetes may be more apparent under mechanical loading. In patients with plantar fasciitis, an ultrasound-based study reported increased plantar fascia thickness, changes in the plantar fascia/fat pad ratio, and reduced fat pad thickness at different measurement points (Çatal et al., 2026). In addition, a systematic review reported that ultrasound is increasingly used to assess plantar fascia thickness in patients with plantar fasciitis (Mohseni-Bandpei et al., 2014b). Plantar fascia thickness may also vary according to individual characteristics, as previous sonographic studies have shown that it increases with age and BMI (Jha et al., 2023), and that overweight and obese individuals present greater plantar fascia and heel pad thickness than normal-weight individuals (Taş et al., 2017; Jha et al., 2023). Similarly, anthropometric variables have been investigated in relation to plantar fascia thickness in healthy asymptomatic subjects, supporting the need to consider baseline individual characteristics when interpreting ultrasound measurements (Pascual Huerta and Alarcón García, 2007). Finally, imaging studies have shown that plantar fascia abnormalities are not limited to fasciitis, since plantar fibromatosis, plantar fascia tears, xanthoma, foreign-body reactions, plantar infections, and diabetic fascial disease may also affect the plantar fascia on imaging (Draghi et al., 2017b). Therefore, plantar fascia thickness should be interpreted as a non-specific ultrasound finding and should be considered within the broader clinical context, rather than as an isolated marker specifically attributable to diabetes.

Age differences between groups should also be taken into account as an important biological factor in connective tissue changes. It has been reported that AGEs accumulate with age in collagen-rich structures due to non-enzymatic reactions between sugars and proteins. This accumulation promotes the formation of abnormal cross-links within collagen fibrils, which reduces their elasticity and increases tissue stiffness (Hjerrild et al., 2019). Consistent with these mechanisms, there were statistically significant differences in age between groups, which may have partially influenced the findings observed in plantar tissues.

Studies have suggested that anthropometric variables such as body weight and BMI may influence plantar fat pad thickness, with statistically significant associations that could be explained by a greater amount of systemic adipose tissue (Maemichi et al., 2020). However, the present study did not fully align with these findings as the variable showing significant differences between diabetic and control participants was height (*p <* 0.010), whereas plantar fat tissue thickness did not exhibit relevant variations. Expected anthropometric differences were identified between men and women, with significantly greater height and body weight values in males. These differences reflect normal sex-related differences and should be taken into account when interpreting the ultrasound results.

This study has several limitations that should be considered. The manual application of pressure during ultrasound measurements may have introduced variability, despite efforts to standardize the procedure. Only intra-observer reliability was assessed, and inter-observer reliability was not evaluated, which may limit the generalizability of the findings. Although the sample size was adequate, the cross-sectional design does not allow for causal inferences. In addition, although several potential confounding factors were controlled through the inclusion and exclusion criteria, some conditions that may influence plantar fascia thickness were not systematically assessed. These included subclinical plantar fasciopathy, inflammatory enthesitis, previous overuse injuries, foot posture abnormalities, occupational standing time, physical activity level, and obesity-related mechanical loading. Therefore, these unmeasured factors should be acknowledged as potential limitations, as they may have influenced plantar fascia thickness and should be considered in future studies.

Based on the results, several directions for future research could further deepen our understanding of plantar tissue characteristics. It would be of interest to evaluate the compressibility of the plantar fat pad and plantar fascia using a dynamometer coupled to the ultrasound probe. This would help to quantify tissue deformation under standardized pressure and analyze its potential relationship with clinical and functional parameters.

It would also be relevant to design and validate a standardized ultrasound protocol for assessing the plantar fascia and heel fat pad, which could improve reproducibility and interprofessional reliability. Another important direction for research is the relationship between different foot morphotypes (high-arched, flat, and neutral) and plantar tissue behavior under static and dynamic conditions. Inertial measurement units (IMUs) and advanced gait analysis techniques could be used to explore this issue. Finally, evaluating plantar fat pad behavior at different walking and running speeds could provide valuable information to determine whether functional thresholds exist beyond which plantar cushioning capacity becomes compromised, which could have potential clinical implications for injury prevention.

## 5 Conclusions

Ultrasound demonstrated high intra-observer reliability, supporting its use for evaluating the heel plantar fat pad and plantar fascia. Patients with cT2DM showed no statistically significant differences in plantar fat pad thickness under loaded or unloaded conditions. However, significant differences were initially identified in plantar fascia thickness between the diabetic and control groups in both conditions. After adjusting for age and height through linear regression analysis, this association remained statistically significant only under loaded conditions. These findings suggest a possible load-dependent association between cT2DM and plantar fascia thickness in the present study. Baseline differences were observed between groups, with participants with diabetes being older, as aging is associated with increased accumulation of AGEs. Expected anthropometric differences were found between sexes, with men having greater height and body weight, which should be considered as baseline characteristics when interpreting the findings.

## Data Availability

The raw data supporting the conclusions of this article will be made available by the authors, without undue reservation.
